# Networks and clusters of immunometabolic biomarkers and depression-associated features in middle-aged and older community-dwelling US adults with and without depression

**DOI:** 10.1016/j.bbih.2025.101103

**Published:** 2025-09-17

**Authors:** Asma Hallab, The Health and Aging Brain Study (HABS-HD) Study Team, HABS-HD MPIs, Sid E. O'Bryant, Kristine Yaffe, Arthur Toga, Robert Rissman, Leigh Johnson, HABS-HD Investigators, Meredith Braskie, Kevin King, James R. Hall, Melissa Petersen, Raymond Palmer, Robert Barber, Yonggang Shi, Fan Zhang, Rajesh Nandy, Roderick McColl, David Mason, Bradley Christian, Nicole Phillips, Stephanie Large, Joe Lee, Badri Vardarajan, Monica Rivera Mindt, Amrita Cheema, Lisa Barnes, Mark Mapstone, Annie Cohen, Amy Kind, Ozioma Okonkwo, Raul Vintimilla, Zhengyang Zhou, Michael Donohue, Rema Raman, Matthew Borzage, Michelle Mielke, Beau Ances, Ganesh Babulal, Jorge Llibre-Guerra, Carl Hill, Rocky Vig

**Affiliations:** a Psychiatry Neuroimaging Laboratory – Psychiatry and Radiology Departments – Mass General Brigham, Harvard Medical School, Boston, MA, USA; b Biologie Intégrative et Physiologie (BIP) – Parcours Neurosciences. Faculté des Sciences et Ingénierie, Sorbonne Université, Paris, France; c Pathologies du Sommeil. Faculté de Médecine Pitié-Salpêtrière, Sorbonne Université, Paris, France

**Keywords:** Low-grade inflammation, Sickness behavior, Anhedonia, Motivation, Melancholia, Immunometabolic depression

## Abstract

**Introduction:**

Therapy-resistant depression is associated with higher levels of systemic inflammation and increased odds of metabolic disorders. It is, therefore, crucial to identify the biomarkers of high-risk individuals and understand the key features of depression-immunometabolic networks.

**Methods:**

The multiethnic ≥50-year-old study population is a subset of the Health and Aging Brain Study: Health Disparities (HABS-HD) study. Spearman's rank correlation network analysis was performed between immunological, metabolic, and subscales of the Geriatric Depression Scale (GDS). Significant correlations were then evaluated using a multivariable linear regression analysis, including testing for non-linearity and clinical cutoffs.

**Results:**

Two clusters were formed: the first included the immunometabolic biomarkers, and the second included the different subscales of GDS. The two clusters were significantly correlated at six edges. IL-6 and HbA1c were significantly correlated with anhedonic and melancholic features. Abdominal circumference and BMI were significantly correlated with anhedonic features. In the subgroup without current depression, IL-6 and Abdominal circumference maintained a significant edge with anhedonic features. TNF-alpha/melancholia and IL-6/cognitive concerns were additional relevant edges in older adults. The observed correlations remained statistically significant in the confounder-adjusted regression analysis and followed specific patterns.

**Conclusions:**

Symptom clustering showed its superiority over relying on dichotomized depression diagnoses for identifying relevant immunometabolic biomarkers. This study is a first step toward understanding the particularities of immunometabolic depression for better risk stratification and to direct personalized preventive and therapeutic strategies in multiethnic aging populations.

## Highlights

• IL-6 and HbA1c were significantly associated with anhedonic and melancholic features.
• Abdominal circumference and BMI were significantly associated with anhedonic features.
• In the subgroup without current depression, IL-6 and Abdominal circumference were correlated with anhedonic features.

## Graphical abstract

**Figure:**
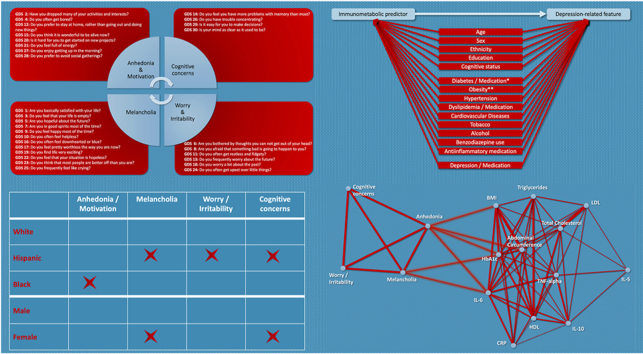

## 1 Introduction

Depression ranks among the top mental health disorders worldwide and is associated with high morbidity and mortality rates (Patwardhan et al., 2024; GBD 2019 Mental Disorders Collaborators, 2022). In addition to its related high health costs, one-third of patients with major depressive disorder show a lack of response to adequate treatment strategies and remain therapy-resistant (Chan et al., 2024). This non-remission predicts further health complications (Chan et al., 2023) and social adversities, including unemployment (Arena et al., 2023) and higher suicide risks (Rhee et al., 2024; Bergfeld et al., 2018; Chan et al., 2022). Obesity and metabolic disorders are prevalent in patients with depression, particularly in severely affected ones and particularly non-responders (Woo et al., 2016). While the effect of depression on metabolic disorders has for long been intuitively associated with the lack of a healthy lifestyle (Wang et al., 2025) and use of antidepressant medication (Moss et al., 2025), recent studies have shown that the causal association is bidirectional (Luppino et al., 2010), and more complex mechanisms are involved. Low-grade inflammation is commonly described in depression and metabolic disorders (Penninx et al., 2025; Hallab, 2025), reflecting a latent state of chronic distress and its concomitant impact on the brain (Hallab, 2025) and peripheral organs, including the adipose tissue (Hallab et al., 2025). Our previous study showed that pro-inflammatory cytokines and metabolic biomarkers were significantly higher in middle-aged and older adults with depression (Hallab, 2025). These findings enabled the integration of the immunological biomarkers, primarily Interleukin-6 (IL-6), as statistical mediators within the depression-metabolism crosstalk (Hallab, 2025). A few recent clinical trials investigated causal associations by exploring new treatment pathways targeting anti-inflammatory molecules and evaluating their added effect in treatment-resistant patients with low-grade inflammation (Gong et al., 2025; Treadway et al., 2025). Besides these controversial results, current literature is limited to descriptive and associative studies, and there is a need to understand the underlying pathophysiology by identifying specific clinically relevant features in high-risk individuals, enabling personalized therapeutic strategies. The translation from a generalized paradigm englobing depression into a single diagnostic entity to acknowledging within- and between-individual variability and contesting the potential immunometabolic-symptom specificity allowed initial steps toward understanding the importance of clustering patient subgroups and initiating individualized approaches (Treadway et al., 2025).

Previous studies dedicated to understanding immune-metabolic networks were explicitly restricted to populations with European ancestry (Kappelmann et al., 2021a). As such, available findings cannot be generalized to multiethnic populations. Ethnic minorities are exposed to higher adversities and consequently increased risks of affective and stress-related disorders (Elhabashy et al., 2025), in addition to higher prevalence of cardiometabolic and immune-mediated pathologies (Hallab et al., 2025). However, these high-risk groups are less represented in clinical studies (O'Bryant et al., 2021). Furthermore, most published studies included broad age ranges, predominantly young and middle-aged populations (Kappelmann et al., 2021a; Fried et al., 2020; Suneson et al., 2023). However, depression-related symptoms and immunometabolism undergo the dynamic influence of aging and immunosenescence (Kivimäki et al., 2024), and combined findings in the young and middle-aged populations might not apply to older adults.

Overall, there is a serious lack of studies on older populations with a multiethnic background. To fill this gap, this study aimed to assess the associations between depression-related features and immunometabolic biomarkers in a multiethnic US population of community-dwelling middle-aged and older adults with and without depression.

## 2 Methods

### 2.1 Study population

The study population is a subset of the Health and Aging Brain Study: Health Disparities (HABS-HD) study, initiated in 2017, to assess health outcomes in Mexican Americans compared to White Americans (O'Bryant et al., 2021). It was expanded in 2020 by including Black American participants. Community-dwelling middle-aged and older adults 50 years and older were recruited at the Institute of Translational Medicine at the University of North Texas Health Science Center (UNTHSC) (Petersen et al., 2025). The follow-up had a 24-30-month rhythm.

Since ethnic minorities tend to be rarely involved or minimally represented in clinical studies, the current cohort recruited participants in community-based events and was advertised in social media and newspapers. Participants were eligible if they were at least 50 years old, had no prior diagnosis of dementia (except Alzheimer's disease), had no Type 1 Diabetes Mellitus (T_1_DM), and have no active cancer during the study or the last 12 months. Furthermore, participants with substance use disorders or severe mental health conditions (except depression and anxiety) were not eligible. Chronic conditions that might impact the cognitive status (exp., late stages of chronic kidney disease, chronic heart failure …) or a history of severe traumatic brain injury were considered exclusion criteria. Participants underwent clinical, neuropsychological, biological, and neuroimaging investigations. The investigations related to the medical history were based on interviews performed by certified specialized clinicians, and participants were asked to disclose current diagnoses and medications (Petersen et al., 2025). Owing to the community-based aspect of the study, no clinical health records were reviewed.

All procedures contributing to this work comply with the ethical standards of the relevant national and institutional committees on human experimentation and the Helsinki Declaration of 1975, as revised in 2013. The local ethics committee at North Texas provided ethical approval for the source study. Written informed consent was signed by all participants included in the cohort. The current research was performed in compliance with the data use agreement and assessed de-identified aggregated data.

### 2.2 Included variables

#### 2.2.1 Medical history

As previously mentioned, the medical history, current medications, and current relevant diagnoses were collected through interviews performed by certified clinicians, including mental health professionals.

Blood pressure was measured twice at rest. The included systolic and diastolic blood pressure values (SBP and DBP) were calculated as the mean of the first and second systolic and diastolic blood pressure measurements, respectively. Obesity was defined as a body mass index (BMI = Weight (Kg)/Height (m)^2^) ≥ 30.

Cardiovascular disorders, hypertension, dyslipidemia, and T_2_DM diagnostic criteria were detailed in previous publications (Hallab, 2025). In short, diagnoses were assigned by certified clinicians based on disclosed medical history during the interview, current medication, or abnormal clinical and/or biological findings (O'Bryant et al., 2021).

The list of medications was screened case by case to identify antidepressant medication (divided into major groups), benzodiazepines (any type), statins (any type), anti-inflammatory drugs (divided into major groups), and anti-diabetes medication (divided into major groups). One case disclosed a non-specific type of antidepressant medication and was therefore not accounted for.

• **Antidepressant medication** was classified into:
- Selective Serotonin Reuptake Inhibitors (SSRIs): Escitalopram, Citalopram, Sertraline, Fluoxetine, Paroxetine.
- Serotonin-Norepinephrine Reuptake Inhibitors (SNRIs): Duloxetine, Venlafaxine, Desvenlafaxine.
- Norepinephrine-Dopamine Reuptake Inhibitors (NDRIs): Bupropion.
- Tricyclic Antidepressants (TCAs): Doxepin, Amitriptyline.
- Serotonin Antagonist and Reuptake Inhibitors (SARIs): Trazodone.
- Noradrenergic and Specific Serotonergic Antidepressant (NaSSA): Mirtazapine.
• **Anti-inflammatory medications** were classified into:
- All non-steroidal anti-inflammatory drugs (NSAIDs).
- All systemic corticoids (unless details on the administration route are missing).
- All disease-modifying anti-rheumatic drugs (DMARDs) (for rheumatological and systemic disorders, and including a few cases with post-transplantation medication).
• **Anti-diabetes medications** were classified into:
- All oral medications.
- All insulin types.

Medications were reported independently of doses, time, and frequency of intake.

#### 2.2.2 Biological biomarkers

Fasting blood was collected and assessed at the same site. The immunological quantifications of cytokines were performed using the Single Molecule Array (SIMOA) technology developed by Quanterix™, which is a highly sensitive method. C-reactive Protein (CRP) concentrations were measured using an electrochemiluminescent immunoassay on the Meso Scale Discovery (MSD)® platform.

The immunological variables were: Interleukin-5 (IL-5) (pg/mL), Interleukin-6 (IL-6) (pg/mL), Interleukin-10 (IL-10) (pg/mL), Tumor-Necrosis Factor (TNF)-alpha (pg/mL), and CRP (mg/mL).

The metabolic variables were: Triglycerides (mg/dL), Total Cholesterol (mg/dL), High-Density-Lipoprotein (HDL) Cholesterol, Low-Density Lipoprotein (LDL) Cholesterol, HbA1c (%), Abdominal circumference (inch), and BMI.

HbA1c (%) has a strong predictive value on the variations of Glucose (mg/dL) levels over the previous weeks and was therefore preferred for the analysis.

#### 2.2.3 Psychological assessment

Participants underwent neuropsychological tests, including the Geriatric Depression Scale (GDS) (Yesavage et al., 1982), administered and analyzed by certified Neuropsychologists.

In this study, for a better interpretation of associations, GDS items were clustered into four clinically relevant subscales:

• **Anhedonia/lack of motivation**: GDS 2 + GDS 4 + GDS 12 + GDS 15 + GDS 20 + GDS 21 + GDS 27 + GDS 28.
• **Melancholia/Negative emotions and cognitions**: GDS 1 + GDS 3 + GDS 5 + GDS 7 + GDS 9 + GDS 10 + GDS 16 + GDS 17 + GDS 19 + GDS 22 + GDS 23 + GDS 25.
• **Worry/Irritability**: GDS 6 + GDS 8 + GDS 11 + GDS 13 + GDS 18 + GDS 24.
• **Cognitive concerns**: GDS 14 + GDS 26 + GDS 26 + GDS 29 + GDS 30.

This approach allows for a better comparability of the results with previous publications.

A semantic clustering was preferred over Exploratory Factor Analysis (EFA) and Principal Component Analysis (PCA) since these were rather data-driven and did not allow for a clinically meaningful clustering of GDS items (Example in Supplementary Table 1).

Both cases with and without depression were eligible for the study. First, to increase the sample size and statistical power of the results. Second, to adjust for the specific effect of depression and medication in comparison to cases without current depression.

Cases were classified into three groups:

• **Current depression with medication:** Participants disclosed having depression during the interview and are under antidepressant medication.
• **Current depression without medication:** GDS total score ≥10 points and no concomitant antidepressant medication.
• **No (current) depression:** GDS total score <10 points, no disclosed depression, with or without antidepressant medication (in a few cases indicated in sleep disorders in this subgroup).

### 2.3 Statistical analyses

#### 2.3.1 Data distribution

The distribution of the variables was visualized using Q-Q plots and plotted histograms overlaid with corresponding density curves. Normality was statistically assessed using the Shapiro-Wilk test. Skewed variables were log-scaled. Nonparametric methods and dichotomization were applied in cases with persistent skewness.

#### 2.3.2 Missing variables

GDS items were reported as binary variables. Individual responses missing completely at random and corresponding to less than 1 % of cases were imputed with the null value. GDS subscales were calculated and reported as continuous variables. No imputation was indicated for the correlation analysis, as this was performed pairwise between available values. Values of variables introduced as confounders in the regression analysis were imputed by the null or median, if they were missing completely at random and presented less than 1 % of cases.

#### 2.3.3 Group comparison

The Mann-Whitney, Kruskal-Wallis, and Chi-squared tests were used to compare groups. Results of test statistics and *p-*values were reported.

#### 2.3.4 Correlations

Spearman's rank correlation was performed pairwise between the immunometabolic biomarkers and the four subscales of GDS as continuous variables. To reduce the risk of Type I error, *p*-values were adjusted for multiple testing using the false discovery rate method (FDR). Statistically significant pairwise correlations (*p*_*FDR*_-value <0.05) were visualized in a heatmap matrix where positive correlations were highlighted in red-spectrum colors, and negative correlations with blue-spectrum colors. Statistically non-significant *p*_*FDR*_-values ≥0.05 were crossed out.

Correlation-based network analysis was performed and visualized. Spearman's coefficient |r|> 0.1 and *p-*value< 0.05 were considered a threshold to visualize relevant statistically significant correlations (|r|≤ 0.1 considered a non-significant correlation). Nodes represented the variables. Edges represented the strength of the correlation and were weighted based on the correlation coefficient value |r|. The distance between the nodes was weighted based on the correlation coefficients. However, the plotting was subject to the constraint of avoiding overlapping and is data- and seed-dependent.

#### 2.3.5 Associative regression analysis

GDS subscales were dichotomized and four dependent variables were considered: “Anhedonia/Lack of motivation” (no = 0/yes = 1), “Melancholia/Negative emotions and cognitions” (no = 0/yes = 1), “Worry/Irritability” (no = 0/yes = 1), and “Cognitive concerns” (no = 0/yes = 1). The dependent variables were calculated as binary outcomes, where having at least one point in the corresponding subscore was attributed a positive value, and having no points in the corresponding subscore (GDS subscore = 0) was attributed the null value.

The independent variables were considered from the immunometabolic cluster. The logistic regression analysis evaluated only variables that survived the adjustment for multiple testing and presented relevant nodes in the correlation-based network analysis, with direct edges to the GDS subscales. GDS subscales without direct correlations with the immunometabolic variables were reported as control groups and for the comparability with previous studies. Independent variables had different distributions; IL-6, HbA1c, and Abdominal circumference were introduced in log-scaled forms to the corresponding models, and BMI was kept in raw form.

Three models were then assessed:

• **Model 1**: adjusted for age (years) + sex (female, male) + ethnicity (“Non-Hispanic White”, “Hispanic”, “Black”) + educational level (years) + and cognitive status (“Normal cognition”, “Mild cognitive impairment”, “Dementia”).
• **Model 2**: Model 1 + cardiovascular diseases (binary) + hypertension (binary) + dyslipidemia-related classes (“No dyslipidemia”, “Dyslipidemia without medication”, “Dyslipidemia with medication”) + T_2_DM-related classes (“No diabetes”, “Diabetes without medication”, “Diabetes with medication”) + use of benzodiazepines (binary) + use of anti-inflammatory medication (“NSAIDs”, “Corticoids”, “DMARDs”) + Alcohol consumption (binary) + current Tobacco smoking (binary).
• **Model 3**: Model 2 + depression-related classes (“No (current) depression”, “Current depression without medication”, “Current depression with medication”).

Variance inflation factor (VIF) was calculated in each model, and only variables with factors around one were included. Furthermore, to reduce the risk of multicollinearity:

• For models where a T_2_DM-related predictor (HbA1c) was introduced as an independent variable, T_2_DM was not included as a confounder.
• For models where the obesity-related predictors (BMI and Abdominal circumference) were introduced as independent variables, obesity was not included as a confounder.

Models 3 were considered the model of choice and visualized, as they accounted for the effects of depression and antidepressant medication in the total population.

Models were assessed for non-linearity along three degrees of freedom (df = 3, 4, and 5), and were compared to the linear model. The Akaike Information Criterion (AIC) was used to find the best fit.

To facilitate the interpretation of the findings, the analyses were repeated based on clinical cutoffs (HbA1c, Abdominal circumference, and BMI). IL-6 was not included in this analysis since levels were predominantly much lower than clinical cutoffs. The dichotomized odds ratios were based on model 3, and the reference group included those with levels under the corresponding cutoff value of the studied variable.

#### 2.3.6 Sensitivity analysis

To optimize the interpretability of intricate findings and ensure the comparability of the results with published data, the tertile categorization was then applied, and the previous steps were repeated with the first tertile, T1, as reference.

The statistical analysis was performed using RStudio (Version, 2024.12.1, Posit®, USA).

## 3 Results

### 3.1 Study data

Ambiguous BMI values (BMI <15 and BMI >70) were considered missing in three cases, particularly for the correlation analysis. These and other missing BMI values were then imputed with the median value, allowing an optimal integration of obesity as a confounder in the regression analysis. Similarly, missing cognition-related diagnoses were imputed by the null in nine cases. The cytokines, CRP, HbA1c, serum lipids, and Abdominal circumference had a right-skewed distribution. BMI had a close-to-normal distribution and was kept in its raw form.

### 3.2 Study population

The study included 3,838 participants aged between 50 and 90 years, with a highly represented multiethnic background (White 35 %, Hispanic 37 %, and Black 28 %). Females presented 62 % of the cohort. The particularities of the study population are detailed in Table 1.

**Table 1:** Characteristics of study population.

Demographical characteristics	Available values	Missing values	Median (IQR); n (%)
Age (years)	3,838	0	65 (59, 71)
Sex (female)	3,838	0	2,379 (62 %)
Ethnicity	3,838	0	
White			1,348 (35 %)
Hispanic			1,425 (37 %)
Black			1,065 (28 %)
Retired	3,822	16 (0.4 %)	2,043 (53 %)
Education (years)	3,838	0	14.0 (12.0, 16.0)
**Cardiometabolic comorbidities**			
Hypertension	3,838	0	2,528 (66 %)
Diabetes	3,838	0	981 (26 %)
Dyslipidemia	3,838	0	2,812 (73 %)
Cardiovascular diseases	3,838	0	278 (7.2 %)
Obesity	3,811	27 (0.7 %)	1,840 (48 %)
Metabolic syndrome	3,751	87 (2.3 %)	2,860 (76 %)
**Physical examination**			
Body mass index	3,811	27 (0.7 %)	30 (26, 34)
Abdominal circumference (inch)	3,824	14 (0.4 %)	40 (36, 45)
Mean systolic blood pressure (mmHg)	3,789	49 (1.3 %)	134 (122, 147)
Mean diastolic blood pressure (mmHg)	3,789	49 (1.3 %)	83 (76, 90)
**Biological biomarkers**			
Total cholesterol levels (mg/dL)	3,679	159 (4.1 %)	180 (153, 208)
Triglycerides levels (mg/dL)	3,678	160 (4.1 %)	107 (77, 149)
High-density lipoprotein cholesterol levels (mg/dL)	3,678	160 (4.1 %)	53 (44, 64)
Low-density lipoprotein cholesterol levels (mg/dL)	3,631	207 (5.4 %)	100 (78, 125)
Glycated hemoglobin A1c levels (%)	3,682	156 (4 %)	5.60 (5.30, 6.10)
Plasma Interleukin-5 (pg/mL)	2,135	1,703 (44.4 %)	0.09 (0.06, 0.15)
Plasma Interleukin-6 (pg/mL)	2,189	1,649 (43 %)	0.94 (0.63, 1.42)
Plasma Interleukin-10 (pg/mL)	2,183	1,655 (43.1 %)	0.35 (0.27, 0.45)
Plasma Tumor necrosis factor-alpha (pg/mL)	2,113	1,725 (45 %)	1.54 (1.26, 1.94)
C-reactive protein (mg/dL)	1,619	2,219 (57.8 %)	9 (2, 48)
**Neuropsychiatric comorbidities**			
Cognitive status	3,838	0	
Healthy controls			2,799 (73 %)
Mild cognitive impairment			803 (21 %)
Dementia			236 (6.1 %)
Anxiety	3,838	0	668 (17 %)
Depression	3,838	0	1,041 (27 %)
Depression classes	3,838	0	
No (current depression)			2,797 (73 %)
Current depression without medication			582 (15 %)
Current depression with medication			459 (12 %)
**Medication**			
Use of antidepressant medication	3,838	0	642 (17 %)
Classes of antidepressant medications	642	0	
SSRIs			377 (59 %)
SSNRIs			98 (15 %)
SARIs			69 (11 %)
NDRIs			56 (8.7 %)
TCAs			26 (4.0 %)
NaSSA			16 (2.5 %)
Number of antidepressant medication (if any)	3,838	0	
One			459 (12 %)
Two or more			183 (4.8 %)
Use of Statins	3,838	0	1,397 (36 %)
Use of antidiabetic medication	3,838	0	698 (18 %)
Use of Benzodiazepines	3,838	0	139 (3.6 %)
Use of anti-inflammatory medication	3,838	0	1,057 (28 %)
Classes of anti-inflammatory medications	3,838	0	
None			2,781 (72 %)
NSAID			1,005 (26 %)
Corticoids			33 (0.9 %)
DMARD			19 (0.5 %)

DMARDs: Disease-modifying antirheumatic drugs, NaSSA: Noradrenergic and Specific Serotonergic Antidepressants, NDRIs: Norepinephrine-Dopamine Reuptake Inhibitors, NSAIDs: Non-steroidal anti-inflammatory drugs, SARIs: Serotonin Antagonist and Reuptake Inhibitors, SSNRIs: Serotonin-Norepinephrine Reuptake Inhibitors, SSRIs: Selective Serotonin Reuptake Inhibitors, TCAs: Tricyclic Antidepressants.

### 3.3 Group comparison

The detailed GDS subscores are detailed in Supplementary Fig. 1. Black participants reported more “Anhedonia/Lack of motivation” symptoms, while Hispanic participants reported more “Melancholia/Negative emotions and cognitions”, “Worry/Irritability”, and “Cognitive concerns” (Fig. 1a). Women reported significantly more “Melancholia/Negative emotions and cognitions” and “Cognitive concerns” compared to males (Fig. 1b).

**Fig. 1:**
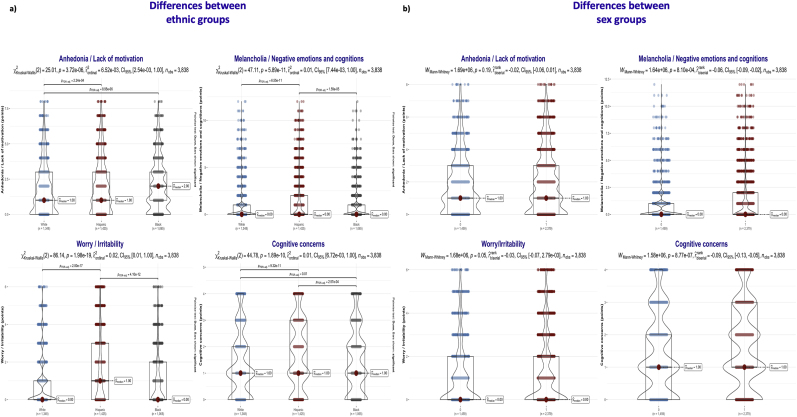
Differences in Geriatric Depression Scale subscores between ethnic and sex groups. **1.a)** Differences between ethnic groups. **1.b)** Differences between sex groups.

**Fig. 2:**
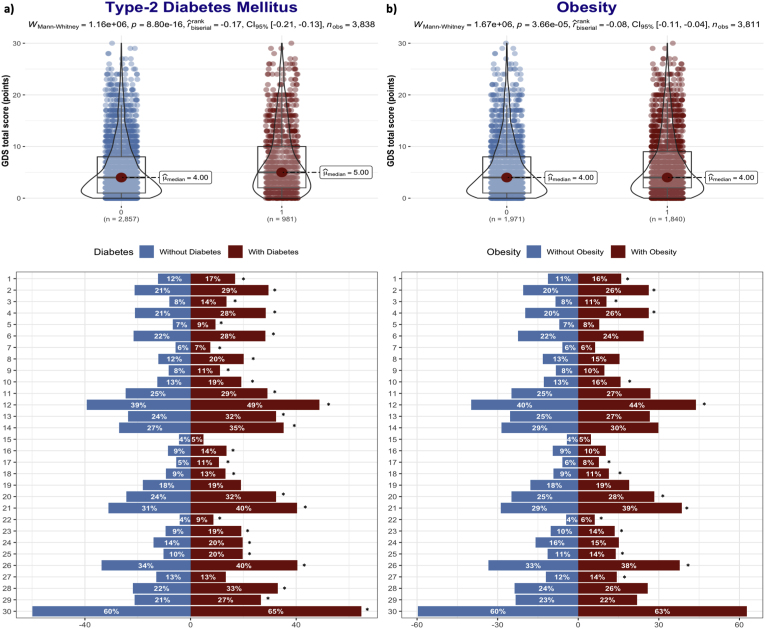
Differences in Geriatric Depression Scale total score and items between participants with and without Type 2 diabetes mellitus and Obesity. **2.a)** Type 2 diabetes mellitus. **2.b)** Obesity. **Footnote:** ∗ for p-value <0.05.

Participants with T_2_DM and obesity had significantly higher GDS scores (*p*-values <0.001). Details on GDS items in T_2_DM and obesity were visualized in Fig. 2.

### 3.4 Correlation analysis

**Fig. 3:**
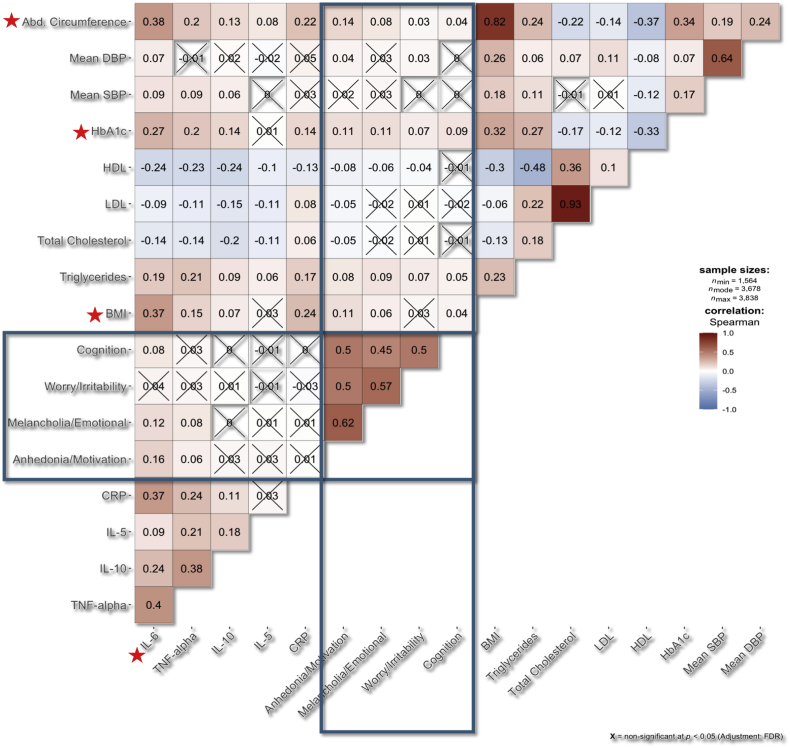
Correlation matrix between immunometabolic biomarkers and GDS subscores with *p*-values adjusted for multiple testing.

**Fig. 4:**
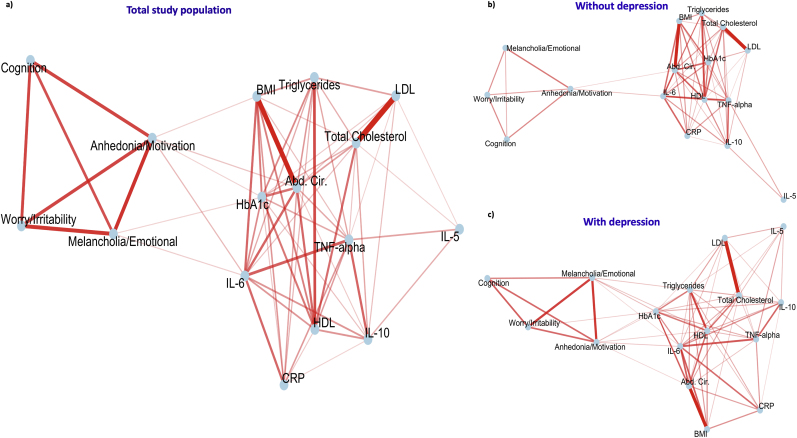
Correlation network analysis in the total study population and stratification by current depression status. **4.a)** Total study population. **4.b)** Without depression. **4.c)** With depression.

A correlation analysis was performed between all relevant variables, and FDR-adjusted results were plotted in a correlation matrix (Fig. 3). In the correlation-based network analysis, two clusters were formed: the first included the immune-metabolic biomarkers, and the second included the different subscales of GDS. The two clusters were significantly correlated at six edges, with four primary nodes of the immunometabolic cluster (IL-6, HbA1c, Abdominal circumference, and BMI) and two nodes of the depression-related features (“Anhedonia/Lack of Motivation” and “Melancholia/Negative emotions and cognition”) (Fig. 4a).

Data were stratified by current depression status. Those without current depression maintained two significant edges between IL-6, Abdominal circumference, and “Anhedonia/Lack of motivation” (Fig. 4b). The number of edges between the two clusters increased in the subgroup with current depression and involved additionally lipids (Fig. 4c).

**Fig. 5:**
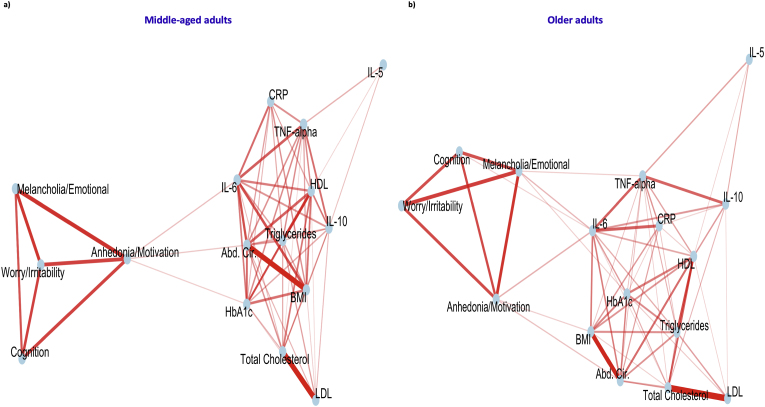
Correlation network analysis stratification by age group. **5.a)** Middle-aged adults. **5.b)** Older adults.

Data were then stratified by age groups. The correlation network in older adults (≥65 years) showed new significant edges between TNF-alpha and “Melancholia/Negative emotions and cognition” and between IL-6 and “Cognitive concerns” (Fig. 5).

### 3.5 Regression analyses

#### 3.5.1 Assessing linearity in regression models

Different models were tested for non-linearity, and the best fit was kept for the analysis.

Higher IL-6 was significantly associated with higher odds of reporting “Anhedonia/Lack of motivation” and “Melancholia/Negative emotions and cognitions” in a linear trend. Similarly, higher Abdominal circumference and BMI measures were linearly associated with higher odds of “Anhedonia/Lack of motivation”.

The HbA1c-“Anhedonia/Lack of motivation” and HbA1c-“Melancholia/Negative emotions and cognitions” associations had non-linear patterns with statistical significance at df = 4. Similarly, the association between Abdominal circumference and “Cognitive concerns” had a non-linear pattern with statistical significance at df = 5 (Supplementary Table 2).

#### 3.5.2 Independent variables based on clinical cutoffs

Clinical cutoff-based models 1, 2, and 3 were detailed in the Supplementary Tables 3, 4, and 5, respectively. HbA1c levels equal to or higher than 6.5 % were significantly associated with higher odds of “Anhedonia/Lack of motivation” (OR = 1.28 [1.02, 1.60], *p*-value = 0.034) and “Melancholia/Negative emotions and cognitions” (OR = 1.27 [1.02, 1.57], *p*-value = 0.030). Abdominal circumference measures equal to or higher than 40 inches for males/35 inches for females, respectively, were associated with higher odds of “Anhedonia/Lack of motivation” (OR = 1.26 [1.06, 1.50], *p*-value = 0.009). Similarly, BMI measures equal to or higher than 30 were associated with higher odds of “Anhedonia/Lack of motivation” (OR = 1.21 [1.03, 1.42], *p*-value = 0.020). The clinical cutoff values of BMI did not explain the association between the variation of BMI and the odds of “Cognitive concerns” at df = 5.

#### 3.5.3 Independent variables based on tertiles

Tertile-based models 1, 2, and 3 were detailed in the Supplementary Tables 6, 7, and 8, respectively.

This method did not outperform the clinical cutoff-based analysis, particularly for HbA1c, as no significant results were observed.

The association between IL-6 and “Anhedonia/Lack of motivation” confirmed the linear trend as OR in T3 (OR = 1.45 [1.12, 1.89], *p*-value = 0.005) and T2 (OR = 1.40 [1.10, 1.79], *p*-value = 0.007) were significantly higher than T1. Only very high values (T3) of Abdominal circumference (OR = 1.30 [1.06, 1.59], *p*-value = 0.010) and BMI (OR = 1.23 [1.01, 1.51], *p*-value = 0.042) were significantly associated with higher “Anhedonia/Lack of Motivation” compared to the reference.

The association between BMI and “Cognitive concerns” showed that values of BMI in T2 were significantly associated with higher odds of “Cognitive concerns” compared to the reference.

**Fig. 6:**
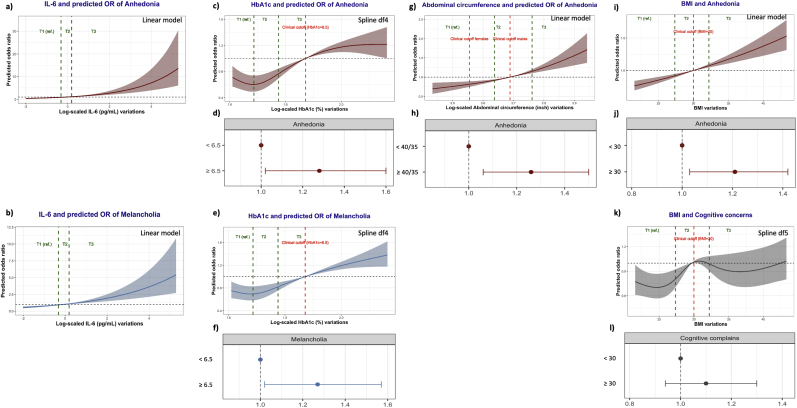
Summary visualization of regression models. **6.a)** IL-6 and predicted odds ratios of anhedonia. **6.b)** IL-6 and predicted odds ratios of melancholia. **6.c)** Glycated hemoglobin A1c and predicted odds ratios of anhedonia. **6.d)** Dichotomized glycated hemoglobin A1c and predicted odds ratios of anhedonia. **6.e)** Glycated hemoglobin A1c and predicted odds ratios of melancholia. **6.f)** Dichotomized glycated hemoglobin A1c and predicted odds ratios of anhedonia. **6.g)** Abdominal circumference and predicted odds ratios of anhedonia. **6.h)** Dichotomized Abdominal circumference and predicted odds ratios of anhedonia. **6.i)** Body mass index and predicted odds ratios of anhedonia. **6.j)** Dichotomized body mass index and predicted odds ratios of anhedonia. **6.k)** Body mass index and predicted odds ratios of cognitive concerns. **6.l)** Dichotomized body mass index and predicted odds ratios of cognitive concerns. **Footnote:** visualization of x-axis values between 5 % and 95 %.

All significant models with plotting of the clinical cutoff-based odds ratio were visualized in Fig. 6.

## 4 Discussion

The main outcome of the study was the two correlation-weighted clusters revealed between immunometabolic variables and different GDS subscores, with four relevant points in the immunometabolic cluster (IL-6, HbA1c, Abdominal circumference, and BMI), all of which created significant direct edges with at least one GDS-specific cluster. Only “Anhedonia/Lack of motivation” and “Melancholia/Negative emotions and cognitions” were directly correlated with the immunometabolic biomarkers in the total population. The stratification by current depression status showed the importance of the correlation between IL-6, Abdominal circumference, and anhedonic features in individuals without current depression. The stratification by age group showed, in older adults, an involvement of TNF-alpha (correlated with melancholia) and cognitive concerns (correlated with IL-6), additionally to the pre-described constellation.

These findings were replicated in the regression analysis after adjusting for multiple confounders. While IL-6 had a linear association with higher odds of anhedonic and melancholic features, clinical cutoffs of other variables showed significant and practically interpretable associations with the same outcomes (“Anhedonia/Lack of motivation” and “Melancholia/Negative emotions and cognitions”). Tertile-based cutoffs allowed the identification of the significant association between medium ranges of BMI and “Cognitive concerns” at df = 5, not revealed by the clinical cutoff-based method. However, the intricate associations with “Cognitive concerns” might be confounded by an intermediate effect of the anhedonic or melancholic features, as visualized in the network analysis, and might not represent direct effects.

### 4.1 Clustering depression

Several strategies were evaluated to assess associations and networks between depression-related symptoms, metabolic, and immunological biomarkers. Netherlands Study of Depression and Anxiety (NESDA) included participants with current or past depression diagnoses (42.9 ± 12.9 years, n = 2,321) and used the self-rated version of the Inventory of Depressive Symptomatology (IDS-SR). The network analysis provided comparable but also conflicting results to our study (Fried et al., 2020). Only IL-6 maintained a direct correlation with the total depression score after adjusting for confounders. In adjusted models where only CRP was evaluated, this was only directly correlated with the “energy level” subscore. Introducing the inflammatory biomarkers in a network with adjusted and regularized models showed no direct association between these and depression subscores. In less conservative adjusted networks without regularization, several inflammation-related edges were statistically significant. The corresponding symptoms were mainly related to sleep, energy, pain, and irritability (Fried et al., 2020). Although sleep and pain are not investigated in GDS items, irritability and worry did not show any significant association with the immunometabolic biomarkers in our study. The association between sleep disorders and dyslipidemia has been commonly described in older populations (Hallab, 2024); however, there is still a need to understand how depression-related sleep disorders, inflammation, and dyslipidemia are associated.

In a German cohort of middle-aged patients with T_1_DM and T_2_DM (44.9 ± 13.9 years, n = 1,260), several immunological biomarkers were tested for their association with different depression features (Herder et al., 2025). Depression symptoms were assessed by the Center for Epidemiological Studies-Depression Scales (CES-D), and the subscores were divided into cognitive-affective, somatic, and anhedonic. The study found significant associations between depression symptoms and some immunological biomarkers; none were included in our study. However, the German study has some limitations, since the exploration of a high panel of immunological biomarkers without adjusting for multiple testing exposes the data to high risks of Type I error. Out of 76 biomarkers, only nine were associated with depression in general, and two to six with each cluster (four with cognitive-affective, six with somatic, and two with anhedonia). The associations were, however, stronger in the T_2_DM than the T_1_DM subgroup after stratification (Herder et al., 2025). In our study, we followed a selective approach based on the correlation network and strength of the correlation coefficients, in addition to adjusting for multiple testing. This allowed focusing on variables directly correlated with GDS subscores. While T_1_DM was not assessed in our cohort, cases with T_2_DM differentiated remarkably in their GDS items from those without diabetes and obesity-related grouping. T_1_DM is less prevalent and has mainly an autoimmunological etiology with a higher cognitive risk profile owing to the severe metabolic fluctuations and the neuro-cerebral complications that develop at an earlier age (Mazzotta et al., 2024). This motivated its exclusion from the source cohort. T_2_DM, however, is more common in middle-aged and older populations, and its onset is mainly related to lifestyle and metabolic comorbidities (Lu et al., 2024).

Older studies tended to classify depression into “melancholic” versus “atypical”. Findings from the Netherlands study on Depression and Anxiety in middle-aged adults (41.3 ± 14.6 years, n = 776) suggested that “melancholic depression” was correlated with hypothalamic-pituitary-adrenal axis hyperactivity (higher cortisol levels), while “atypical depression” was rather correlated with inflammatory and metabolic dysregulations (Lamers et al., 2013). Considering “anhedonia” as the modern label of “atypical depression”, both melancholic and atypical features were associated with the immunometabolic biomarkers in our study, which contradicts the latter study's dichotomization approach.

A large two-sample Mendelian randomization-based study (Kappelmann et al., 2021b) showed a significant association between higher IL-6 signaling and suicidality. No statistically significant association was found between other depressive symptoms and higher CRP levels or IL-6 signaling. The study showed, however, that anhedonia, tiredness, feelings of inadequacy, and changes in appetite were significantly associated with higher BMI (Kappelmann et al., 2021b). Depression symptoms were assessed using the Patient Health Questionnaire-9 (PHQ-9), and the study did not differentiate between cases with and without major depression, both eligible, as in our cohort. However, no age ranges were reported, and it remains unclear whether the results might be replicated in older populations. The study confirms our results regarding the lack of association between CRP levels and depression-specific features in the total study population. However, in our study, measures of the Abdominal circumference were more promising in estimating anhedonic features than BMI when we decreased the number of cases in groups after depression-specific stratification. Furthermore, the contradictory results regarding the association between IL-6 and anhedonia between our study and the latter pose questions about whether anhedonic features captured in our analysis might be a prior stage or proxy of higher risk of suicidal ideation. GDS does not directly explore the presence of suicidal ideas. However, one question (GDS 15: “Do you think it is wonderful to be alive now?”) might present some indirect annotation and was incorporated into the anhedonic features in our study. Furthermore, many questions referring to tiredness and feelings of inadequacy were classified into anhedonic and melancholic features in our study; both clusters were significantly associated with high IL-6.

Combining data from the UK-Biobank and NESDA showed that IL-6 but not CRP was associated with PHQ-9-related anhedonia (Milaneschi et al., 2021). Higher NESDA-measured IL-6 levels were additionally associated with higher odds of hypersomnia, fatigue, and decreased appetite. Higher CRP levels, however, were associated with increased odds of fatigue, sleep problems, depressed moods, and irritability after a meta-analytic pooling of estimates across the two cohorts. Despite maintaining their statistical significance, these latter associations were attenuated after adjusting for BMI. In the Mendelian Randomization analysis, genetically predicted higher CRP levels were associated with suicidal ideation and cognitive problems. The genetically predicted high IL-6 signaling was associated with fatigue and sleep alterations (Milaneschi et al., 2021).

A network analysis-based study on polygenic risk scores of immunometabolic biomarkers and depression symptoms showed an association between CRP and changes in appetite, fatigue, and anhedonia (Kappelmann et al., 2021a). Contrary to previous findings, CRP and anhedonia exhibited a negative association. Furthermore, the study showed a significant association between TNF-alpha and fatigue and between BMI, anhedonia, and changes in appetite (Kappelmann et al., 2021a). Despite the interesting findings, the study has several limitations. The combination of clinical and population-based cohorts, the use of different assessment tools for depression symptoms across different cohorts (Hamilton Rating Scale for Depression and PHQ-9), the lack of IL-6 and IL-10 specific assessments in one of the included cohorts (selection bias), and, as previously mentioned, the restrictiveness to participants with European ancestry present major limitations. The inclusion of younger age groups (mean ages: 47.8, 43.2, and 56.2 years) limits the generalizability of the results to older populations (Kappelmann et al., 2021a).

Clustering patients based on CRP levels and “difficult-to-treat depression” criteria in a clinical study (37.9 ± 13.3, n = 263 with depression) showed higher inflammatory biomarkers (IL-6 and IL8), and higher BMI in those with CRP > 3 mg/L (Suneson et al., 2023) The exclusion of cases with inflammatory disorders did not change the results. However, no significant difference in the Montgomery-Asberg Depression Rating Scale (MADRS) or suicide assessment scale (SUAS) scores was found between the groups with higher and lower CRP levels (Suneson et al., 2023).

### 4.2 Diabetes

The Irish Longitudinal Study on Aging-based analysis in a large cohort of middle-aged and older adults (≥49 years, n = 3,895) showed a significant association between higher CRP levels and depression in T_2_DM participants but not in controls (Huang et al., 2021). Furthermore, only in those with T_2_DM, CRP levels at baseline predicted higher depression risk after a 4-year follow-up (Huang et al., 2021). In a large Japanese cross-sectional study on middle-aged and older adults with T_2_DM (66 ± 11.4 years, n = 3,573), higher CRP levels were associated with 169 % higher odds of having depression only in those with a BMI ≥25 (recommended cutoff for obesity in the Japanese population) (Hayashino et al., 2014). In the same study, stratifying by HbA1c levels did not show significant results (Hayashino et al., 2014). The depression diagnosis was based on nine items from the PHQ-9. The authors did not exclude a potential involvement of insulin resistance in the observed results, and acknowledged the limitation associated with the lack of this information (Hayashino et al., 2014). A smaller study in middle-aged participants (48.1 ± 12 years, n = 219) with poorly controlled diabetes found a significant value of baseline CRP levels in predicting a lower improvement of depression symptoms after treatment in T_1_DM but not T_2_DM (Zahn et al., 2016). The lack of data on IL-6 levels in these studies limits the interpretation of these results, and the differences between populations and severity of diabetes might explain observed discrepancies.

### 4.3 Obesity

Our previous study has shown a strong association between depression, IL-6 levels, and obesity. IL-6 was a strong mediator in the association between depression and obesity (Hallab, 2025). The association between depression, BMI, and inflammation has largely been investigated, and studies have shown conflicting results. A 2009–2010 National Health and Nutrition and Survey (NHANES) based study showed that in 585 participants with depression (48.74 ± 15.88 years), higher CRP levels (3 and 5 mg/L as cutoffs) were significantly associated with higher BMI and higher odds of metabolic syndrome (2.81 and 1.94, respectively) (Rethorst et al., 2014). Depression was assessed using PHQ-9. In smaller UK-based research in middle-aged adults (48.7 ± 15.1 years, n = 406), the associations between the polygenic risk score of depression and IL-10 levels, and the polygenic risk score of BMI, IL-6, and CRP levels did not survive the adjustment for multiple testing (Palmos et al., 2021).

Another NESDA-based study (40.96 ± 12.25 years, n = 1,077) showed a significant association between CRP and atypical, energy-related symptoms (AES = increase in appetite, weight gain, fatigue, hypersomnia, and leaden paralysis) in patients with current major depression (Zwiep et al., 2025). The study found a significant dose-related association between CRP, AES, and different immune-metabolic biomarkers (IL-6, TNF-alpha, Glycoprotein acetyls, BMI, Waist circumference, Triglycerides, Glucose, Leptin, and HDL cholesterol) (Zwiep et al., 2025). While this study adjusted for the use of antidepressant medication, which presents a strength in the analysis, CRP, on which the study was based, is a nonspecific biomarker of inflammation, as the contradictory results of previous studies have shown.

In our study, CRP showed no direct correlation with GDS subscores, which does not exclude significant, but weaker, associations as shown in the correlation matrix. Biologically, IL-6 is a precursor of CRP (Del et al., 2018). This might explain the observed constellation. IL-6 is also locally produced in the brain, and systemic IL-6 might cross the blood-brain barrier (Felger and Treadway, 2017). IL-6 might, therefore, be a better biomarker for anhedonia compared to CRP (Felger and Treadway, 2017), but it does not exclude the utility of CRP in studies where IL-6 is missing (Del et al., 2018). Further studies need to replicate our findings in different populations and age groups.

### 4.4 Clinical implications and perspectives

The association between IL-6, Abdominal circumference, and anhedonic features independently of depression highlights the major role of low-grade inflammation in sickness behavior seen in metabolic disorders and a broad spectrum of neuroinflammatory diseases. The current association in non-depressed individuals might highlight a latent risk or vulnerability to “immunometabolic” depression, and future longitudinal studies need to assess the prospective evolution in this particular subgroup. Another hypothesis might be that anhedonic features/sickness behavior might predict further neuropsychiatric pathologies – other than depression – that need to be better explored in larger prospective population-based studies.

The current study calls for focusing on “first-line” predictors when studying the association between immune-metabolic biomarkers and depression-related features. IL-6, Abdominal circumference, and HbA1c seem to be accurate biomarkers for anhedonia and melancholia in the total study population. This contrasts with lipid profiles, for example, which were only accurate in participants with depression. The study also showed that anhedonic and melancholic symptoms might better predict concurrent immune-metabolic disorders, in contrast with other depression profiles dominated by irritability, worry, and cognitive concerns in middle-aged individuals. Preventive strategies need to incorporate physical health and target metabolic homeostasis (reducing abdominal obesity and increasing physical activity, stabilizing diabetes/prediabetes …) particularly in anhedonic/melancholic individuals.

Future investigations will priorities (A) assessing the prospective risks associated with higher IL-6 levels and larger Abdominal circumference in those without depression and (B) assessing the longitudinal course of depression in those already affected and expressing particularly higher anhedonic and melancholic symptoms.

### 4.5 Strengths

This is the first study to explore the association between the immunometabolic and depression-associated features specifically in middle-aged and older adults of multi-ethnic background. The large number of included participants and the high availability of inflammation biomarkers present a major strength of the current analysis. The studied biomarkers are clinically relevant and might be assessed in everyday practice, and the assessment of IL-5, IL-6, IL-10, and TNF-alpha is a further strength compared to studies that focused only on CRP. The use of the GDS score, a clinically relevant diagnostic tool with high sensitivity in older adults, is a further novelty compared to available data. The application of different methods to highlight the associations between the variables and the exploration and visualization of relationships within and between clusters is a novelty. The inclusion of a large sample of ethnic minorities, with higher risks of stress, depression, and cardiometabolic disorders, in addition to the community-based aspect of the source cohort, allows for more generalizability of the results in ethnically diverse settings of community-dwelling adults. Finally, the current findings were adjusted for multiple confounders not explored in previous publications, mainly the effect of anti-inflammatory, statin, antidepressant, and antidiabetic medications. The findings were also adjusted for the effect of cognitive decline since apathy and anhedonia might be independently reported in the course of dementia (Vellone et al., 2025). This reflects the robustness of the presented associations.

### 4.6 Limitations

The missing values in some immunological biomarkers present a major limitation. CRP levels were included for the comparability of our data with previous publications, but were not assessed in African-American participants. This presents a selection bias toward this variable, and the corresponding results should be interpreted with precaution as they might not apply to African-American populations. Besides CRP, the included number of participants remained significantly high compared to previous cohorts, and correlation analyses were performed pairwise to avoid loss of important information in the data. The community-based aspect of the cohort and the non-random sampling of study participants might present a selection bias by including socially active individuals. However, this recruitment strategy allowed reaching ethnic groups rarely covered by clinical studies and randomized trials. The focus of the current analysis was on community-dwelling middle-aged and older adults with a higher representation of ethnic minorities, and it does not reflect potential findings in more severely affected persons, generally captured in geriatric clinical studies. Furthermore, the median educational level was 14 years, which indicates high participation rates of well-educated individuals, and the study might not reflect findings in more deprived communities. Clinical diagnoses were based on self-disclosure, and no clinical records were revised. However, this might present a very low risk to the significance of our results, seeing that the main analyses and results were based on biological and neuropsychological assessments performed by certified study clinicians. The cross-sectional design is a further limitation, since we cannot draw a causal conclusion from the current results. Subsequent assessments were made at 24–30 months, and many participants were lost to follow-up owing to the COVID pandemic and the non-clinical aspect of the study. The current study focused more on understanding clusters and drawing concomitant networks between them rather than exploring causal associations. Longitudinal frameworks are exposed to both within and between individual variability in the inflammatory and metabolic biomarkers and do not present the best design to answer the current study question, based on associations and not causation. Also, no information is available on prior variations of the variables of interest, and despite adjusting for anti-inflammatory medication, the data might have not captured some unmeasured acute inflammatory states. Finally, the current findings might not apply to participants with T_1_DM and the observed networks are drawn based on specific variables measured in our population. These might not be reproduced in different non-US populations with lower immunometabolic disorders or individuals with lower stress and depression rates than ethnic minorities.

## 5 Conclusions

This correlation network-based analysis of a large multiethnic middle-aged and older population is in favor of an overlap between immunological, metabolic, and specific depression-related features. These features are neither specific nor restricted to depression, as the correlations between IL-6, Abdominal circumference, and anhedonic features were maintained in the subgroup without current depression. This study is a first step toward clustering different subtypes of immunometabolic depression-associated symptoms for a better risk stratification and to direct personalized preventive and therapeutic strategies.

## CRediT authorship contribution statement

**Asma Hallab:** Writing – review & editing, Writing – original draft, Visualization, Validation, Supervision, Software, Project administration, Methodology, Investigation, Formal analysis, Data curation, Conceptualization. Members of the HABS-HD are listed as a group author to acknowledge the collection and provision of the data used in this study, as well as the funding acquisition.

## Ethical approval

All procedures contributing to this work comply with the ethical standards of the relevant national and institutional committees on human experimentation and with the Helsinki Declaration of 1975, as revised in 2013. Ethical approval was obtained from the local institutional review board. Participants gave written informed consent. The current research is based on a secondary analysis of anonymized data and was performed in compliance with the data use agreement (DUA with Hallab).

## Authorization for publication

The principal investigator and data administrator of the HABS-HD study reviewed the manuscript for its compliance with DUA and authorized the submission and publication of the current version.

## Clinical trial number

Not applicable.

## Funding

“Research reported on this publication was supported by the 10.13039/100000049National Institute on Aging of the 10.13039/100000002National Institutes of Health under Award Numbers R01AG054073, R01AG058533, R01AG070862, P41EB015922, and U19AG078109. The content is solely the responsibility of the authors and does not necessarily represent the official views of the 10.13039/100000002National Institutes of Health.” AH did not receive any specific grant from funding agencies in the public, commercial, or not-for-profit sector for this work. Open Access funding is supported by "Read & Publish" agreement between Sorbonne University - Paris and Elsevier.

## Declaration of competing interest

None. The authors have nothing to declare.

## Figures and Tables

**figs1:**
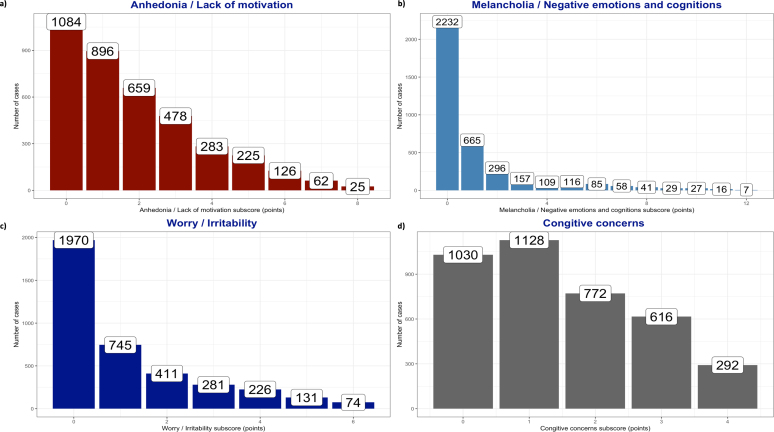


## Data Availability

Data can be acquired by qualified researchers after an official request (asma.hallab@charite.de).
