# Supplementary material for: Networks and clusters of immunometabolic biomarkers and depression-associated features in middle-aged and older community-dwelling US adults with and without depression
Source: Brain Behav Immun Health. 2025 Sep 17;49:101103. doi: 10.1016/j.bbih.2025.101103 (PMC12523063; doi:10.1016/j.bbih.2025.101103)
Supplement: Multimedia component 1 [file mmc1.docx]

**Supplementary Table 1:** Example of Exploratory Factor Analysis with four clusters

|  | **Assigned_Factor** |
| --- | --- |
| GDS 1 | 1 |
| GDS 2 | 1 |
| GDS 3 | 1 |
| GDS 4 | 1 |
| GDS 5 | 1 |
| GDS 6 | 1 |
| GDS 7 | 1 |
| GDS 8 | 1 |
| GDS 9 | 1 |
| GDS 10 | 1 |
| GDS 11 | 1 |
| GDS 12 | 1 |
| GDS 13 | 1 |
| GDS 14 | 1 |
| GDS 15 | 1 |
| GDS 16 | 1 |
| GDS 17 | 1 |
| GDS 18 | 1 |
| GDS 19 | 1 |
| GDS 20 | 1 |
| GDS 21 | 1 |
| GDS 22 | 1 |
| GDS 23 | 1 |
| GDS 24 | 1 |
| GDS 25 | 1 |
| GDS 26 | 1 |
| GDS 27 | 1 |
| GDS 28 | 1 |
| GDS 29 | 1 |
| GDS 30 | 1 |
| Log IL-6 | 3 |
| Log IL-5 | *NA* |
| Log IL-10 | *NA* |
| Log TNF-alpha | *NA* |
| Log CRP | *NA* |
| Log Abdominal Circumference | 3 |
| BMI | 3 |
| Log Triglycerides | 4 |
| Log Total Cholesterol | 2 |
| Log HDL | 4 |
| Log LDL | 2 |
| Log HBA1c | *NA* |
