# Supplementary material for: Networks and clusters of immunometabolic biomarkers and depression-associated features in middle-aged and older community-dwelling US adults with and without depression
Source: Brain Behav Immun Health. 2025 Sep 17;49:101103. doi: 10.1016/j.bbih.2025.101103 (PMC12523063; doi:10.1016/j.bbih.2025.101103)
Supplement: Multimedia component 2 [file mmc2.docx]

**Supplementary Table 2:** Model performance

|  | Anhedonia and lack of motivation | Melancholia and negative emotions/cognitions | Worry and irritability | Cognitive complains |
| --- | --- | --- | --- | --- |
| Log-scaled IL- 6 | | | | |
| Linear model | **p_linear_ <0.0001*** | **p_linear_=0.036*** | p_linear_=0.559 | p_linear_=0.202 |
| df=3 | p_non-linear_=0.949 | p_non-linear_=0.493 | p_non-linear_=0.259 | p_non-linear_=0.904 |
| df=4 | p_non-linear_=0.188 | p_non-linear_=0.785 | p_non-linear_=0.310 | p_non-linear_=0.973 |
| df=5 | p_non-linear_=0.061 | p_non-linear_=0.900 | p_non-linear_=0.488 | p_non-linear_=0.523 |
| Log-scaled HbA1c | | | | |
| Linear model | p_linear_=0.145 | **AIC=3775.596, p_linear_=0.040** | p_linear_=0.426 | p_linear_=0.426 |
| df=3 | p_non-linear_=0.095 | p_non-linear_= 0.089 | p_non-linear_=0.865 | p_non-linear_=0.865 |
| df=4 | **AIC=3807.81, p_non-linear_=0.003*** | **AIC=3770.437, p_non-linear_=0.010*** | p_non-linear_=0.644 | p_non-linear_=0.644 |
| df=5 | **AIC=3809.836, p_non-linear_=0.008** | **AIC=3772.14, p_non-linear_=0.023** | p_non-linear_=0.797 | p_non-linear_=0.797 |
| Log-scaled Abdominal circumference | | | | |
| Linear model | **p_linear_=0.0001*** | p_linear_=0.089 | p_linear_=0.523 | p_linear_=0.191 |
| df=3 | p_non-linear_=0.2246 | p_non-linear_=0.906 | p_non-linear_=0.458 | p_non-linear_=0.589 |
| df=4 | p_non-linear_=0.2069 | p_non-linear_=0.348 | p_non-linear_=0.744 | p_non-linear_=0.657 |
| df=5 | p_non-linear_=0.291 | p_non-linear_=0.481 | p_non-linear_=0.865 | p_non-linear_=0.745 |
| Raw BMI | | | | |
| Linear model | **p_linear_=0.001*** | p_linear_=0.390 | p_linear_=0.497 | p_linear_= 0.223 |
| df=3 | p_non-linear_=0.151 | p_non-linear_=0.366 | p_non-linear_=0.949 | p_non-linear_= 0.427 |
| df=4 | p_non-linear_=0.355 | p_non-linear_=0.153 | p_non-linear_=0.830 | p_non-linear_= 0.824 |
| df=5 | p_non-linear_=0.562 | p_non-linear_=0.275 | p_non-linear_=0.945 | **p_non-linear_= 0.043*** |
