# Supplementary material for: Networks and clusters of immunometabolic biomarkers and depression-associated features in middle-aged and older community-dwelling US adults with and without depression
Source: Brain Behav Immun Health. 2025 Sep 17;49:101103. doi: 10.1016/j.bbih.2025.101103 (PMC12523063; doi:10.1016/j.bbih.2025.101103)
Supplement: Multimedia component 3 [file mmc3.docx]

**Supplementary Table 3:** Regression model 1 with independent variables dichotomized in clinically relevant subgroups.

|  | Anhedonia and lack of motivation | Melancholia and negative emotions or cognitions | Worry and irritability | Cognitive complains |
| --- | --- | --- | --- | --- |
| HbA1c | | | | |
| < 6.5 | — | — | — | — |
| ≥ 6.5 | **1.44 (1.17, 1.78), p<0.001** | **1.34 (1.12, 1.60), p=0.001** | 1.13 (0.94, 1.35), p=0.200 | **1.38 (1.12, 1.71), p=0.003** |
| Abdominal circumference | | | | |
| < 40M / 35F | — | — | — | — |
| ≥ 40M / 35F | **1.42 (1.21, 1.66), p<0.001** | **1.32 (1.13, 1.54), p<0.001** | 1.11 (0.95, 1.29), p=0.200 | 1.17 (0.99, 1.38), p=0.062 |
| BMI | | | | |
| < 30 | — | — | — | — |
| ≥ 30 | **1.34 (1.16, 1.55), p<0.001** | 1.12 (0.98, 1.28), p=0.100 | 1.03 (0.90, 1.18), p=0.600 | **1.20 (1.03, 1.39), p=0.017** |
| Models adjusted for age (years) + sex (female, male) + ethnicity (“Non-Hispanic White”, “Hispanic”, “Black”) + educational level (years) + and cognitive status (“Normal cognition”, “Mild cognitive impairment”, “Dementia”). | | | | |
