# Supplementary material for: Networks and clusters of immunometabolic biomarkers and depression-associated features in middle-aged and older community-dwelling US adults with and without depression
Source: Brain Behav Immun Health. 2025 Sep 17;49:101103. doi: 10.1016/j.bbih.2025.101103 (PMC12523063; doi:10.1016/j.bbih.2025.101103)
Supplement: Multimedia component 4 [file mmc4.docx]

**Supplementary Table 4:** Regression model 2 with independent variables dichotomized in clinically relevant subgroups.

|  | Anhedonia and lack of motivation | Melancholia and negative emotions or cognitions | Worry and irritability | Cognitive complains |
| --- | --- | --- | --- | --- |
| HbA1c | | | | |
| < 6.5 | — | — | — | — |
| ≥ 6.5 | **1.33 (1.08, 1.65), p=0.009** | **1.30 (1.08, 1.57), p=0.005** | 1.13 (0.94, 1.36), p=0.200 | **1.31 (1.05, 1.63), p=0.017** |
| Abdominal circumference | | | | |
| < 40M / 35F | — | — | — | — |
| ≥ 40M / 35F | **1.34 (1.14, 1.59), p<0.001** | **1.26 (1.07, 1.48), p=0.006** | 1.11 (0.94, 1.28), p=0.200 | 1.11 (0.94, 1.32), p=0.200 |
| BMI | | | | |
| < 30 | — | — | — | — |
| ≥ 30 | **1.28 (1.10, 1.49), p=0.002** | 1.07 (0.93, 1.23), p=0.400 | 1.02 (0.89, 1.17), p=0.800 | 1.15 (0.99, 1.35), p=0.070 |
| Models adjusted for age (years) + sex (female, male) + ethnicity (“Non-Hispanic White”, “Hispanic”, “Black”) + educational level (years) + and cognitive status (“Normal cognition”, “Mild cognitive impairment”, “Dementia”) + cardiovascular diseases (binary) + hypertension (binary) + dyslipidemia-related classes (“No dyslipidemia”, “Dyslipidemia without medication”, “Dyslipidemia with medication”) + T2DM-related classes (“No diabetes”, “Diabetes without medication”, “Diabetes with medication”) + use of benzodiazepines (binary) + Alcohol consumption (binary) + current Tobacco smoking (binary). | | | | |
