# Supplementary material for: Networks and clusters of immunometabolic biomarkers and depression-associated features in middle-aged and older community-dwelling US adults with and without depression
Source: Brain Behav Immun Health. 2025 Sep 17;49:101103. doi: 10.1016/j.bbih.2025.101103 (PMC12523063; doi:10.1016/j.bbih.2025.101103)
Supplement: Multimedia component 5 [file mmc5.docx]

**Supplementary Table 5:** Regression model 3 with independent variables dichotomized in clinically relevant subgroups.

|  | Anhedonia and lack of motivation | Melancholia and negative emotions or cognitions | Worry and irritability | Cognitive complains |
| --- | --- | --- | --- | --- |
| HbA1c | | | | |
| < 6.5 | — | — | — | — |
| ≥ 6.5 | **1.28 (1.02, 1.60), p=0.034** | **1.27 (1.02, 1.57), p=0.030** | 1.06 (0.86, 1.30), p=0.600 | **1.26 (1.00, 1.58), p=0.048** |
| Abdominal circumference | | | | |
| < 40M / 35F | — | — | — | — |
| ≥ 40M / 35F | **1.26 (1.06, 1.50), p=0.009** | 1.16 (0.96, 1.40), p=0.120 | 1.01 (0.85, 1.19), p>0.900 | 1.04 (0.87, 1.24), p=0.700 |
| BMI | | | | |
| < 30 | — | — | — | — |
| ≥ 30 | **1.21 (1.03, 1.42), p=0.020** | 0.97 (0.82, 1.15), p=0.700 | 0.94 (0.81, 1.10), p=0.400 | 1.10 (0.94, 1.30), p=0.200 |
| Models adjusted for age (years) + sex (female, male) + ethnicity (“Non-Hispanic White”, “Hispanic”, “Black”) + educational level (years) + and cognitive status (“Normal cognition”, “Mild cognitive impairment”, “Dementia”) + cardiovascular diseases (binary) + hypertension (binary) + dyslipidemia-related classes (“No dyslipidemia”, “Dyslipidemia without medication”, “Dyslipidemia with medication”) + T2DM-related classes (“No diabetes”, “Diabetes without medication”, “Diabetes with medication”) + use of benzodiazepines (binary) + Alcohol consumption (binary) + current Tobacco smoking (binary) + depression-related classes (“No (current) depression”, “Current depression without medication”, “Current depression with medication”). | | | | |
