# Supplementary material for: Networks and clusters of immunometabolic biomarkers and depression-associated features in middle-aged and older community-dwelling US adults with and without depression
Source: Brain Behav Immun Health. 2025 Sep 17;49:101103. doi: 10.1016/j.bbih.2025.101103 (PMC12523063; doi:10.1016/j.bbih.2025.101103)
Supplement: Multimedia component 6 [file mmc6.docx]

**Supplementary Table 6:** Regression model 1 with independent variables classified in tertiles.

|  | Anhedonia and lack of motivation | Melancholia and negative emotions or cognitions | Worry and irritability | Cognitive complains |
| --- | --- | --- | --- | --- |
| IL- 6 | | | | |
| T1 | — | — | — | — |
| T2 | **1.51 (1.21, 1.89), p<0.001** | 1.22 (0.98, 1.53), p=0.076 | 1.02 (0.82, 1.26), p=0.900 | 1.10 (0.88, 1.39), p=0.400 |
| T3 | **1.73 (1.38, 2.19), p<0.001** | **1.47 (1.18, 1.84), p<0.001** | 1.17 (0.94, 1.45), p=0.200 | **1.37 (1.08, 1.74), p=0.011** |
| HbA1c | | | | |
| T1 | — | — | — | — |
| T2 | 0.98 (0.82, 1.17), p=0.800 | 1.04 (0.87, 1.24), p=0.700 | 0.99 (0.84, 1.18), p>0.900 | 1.17 (0.97, 1.41), p=0.110 |
| T3 | **1.34 (1.11, 1.61), p=0.002** | **1.29 (1.09, 1.52), p=0.004** | 1.01 (0.85, 1.19), p>0.900 | **1.21 (1.00, 1.46), p=0.045** |
| Abdominal circumference | | | | |
| T1 | — | — | — | — |
| T2 | **1.21 (1.02, 1.45), p=0.029** | **1.28 (1.08, 1.51), p=0.004** | 1.07 (0.91, 1.26), p=0.400 | 1.11 (0.93, 1.33), p=0.300 |
| T3 | **1.55 (1.30, 1.86), p<0.001** | **1.31 (1.11, 1.54), p=0.002** | 1.05 (0.89, 1.23), p=0.600 | **1.28 (1.06, 1.54), p=0.009** |
| BMI | | | | |
| T1 | — | — | — | — |
| T2 | 1.09 (0.91, 1.29), p=0.400 | 1.04 (0.88, 1.23), p=0.600 | 0.95 (0.81, 1.12), p=0.600 | **1.30 (1.08, 1.56), p=0.005** |
| T3 | **1.41 (1.17, 1.69), p<0.001** | 1.11 (0.94, 1.32), p=0.200 | 1.02 (0.86, 1.20), p=0.800 | **1.30 (1.08, 1.56), p=0.006** |
| Models adjusted for age (years) + sex (female, male) + ethnicity (“Non-Hispanic White”, “Hispanic”, “Black”) + educational level (years) + and cognitive status (“Normal cognition”, “Mild cognitive impairment”, “Dementia”). | | | | |
