# Supplementary material for: Networks and clusters of immunometabolic biomarkers and depression-associated features in middle-aged and older community-dwelling US adults with and without depression
Source: Brain Behav Immun Health. 2025 Sep 17;49:101103. doi: 10.1016/j.bbih.2025.101103 (PMC12523063; doi:10.1016/j.bbih.2025.101103)
Supplement: Multimedia component 7 [file mmc7.docx]

**Supplementary Table 7:** Regression model 2 with independent variables classified in tertiles.

|  | Anhedonia and lack of motivation | Melancholia and negative emotions or cognitions | Worry and irritability | Cognitive complains |
| --- | --- | --- | --- | --- |
| IL- 6 | | | | |
| T1 | — | — | — | — |
| T2 | **1.42 (1.13, 1.79), p=0.003** | 1.17 (0.93, 1.47), p=0.200 | 0.99 (0.79, 1.23), p>0.900 | 1.05 (0.83, 1.34), p=0.700 |
| T3 | **1.49 (1.16, 1.91), p=0.002** | **1.31 (1.03, 1.66), p=0.027** | 1.08 (0.86, 1.37), p=0.500 | 1.23 (0.95, 1.60), p=0.110 |
| HbA1c | | | | |
| T1 | — | — | — | — |
| T2 | 0.95 (0.79, 1.15), p=0.600 | 1.06 (0.88, 1.26), p=0.600 | 1.02 (0.86, 1.21), p=0.800 | 1.16 (0.95, 1.40), p=0.140 |
| T3 | **1.22 (1.00, 1.49), p=0.047** | **1.26 (1.05, 1.51), p=0.011** | 1.01 (0.85, 1.21), p=0.900 | 1.13 (0.93, 1.38), p=0.200 |
| Abdominal circumference | | | | |
| T1 | — | — | — | — |
| T2 | 1.17 (0.98, 1.40), p=0.082 | **1.24 (1.04, 1.47), p=0.015** | 1.06 (0.90, 1.25), p=0.500 | 1.08 (0.90, 1.30), p=0.400 |
| T3 | **1.45 (1.20, 1.76), p<0.001** | **1.21 (1.02, 1.45), p=0.030** | 1.03 (0.87, 1.22), p=0.800 | 1.20 (0.99, 1.46), p=0.059 |
| BMI | | | | |
| T1 | — | — | — | — |
| T2 | 1.06 (0.89, 1.27), p=0.500 | 1.02 (0.86, 1.21), p=0.800 | 0.96 (0.81, 1.13), p=0.600 | **1.28 (1.06, 1.54), p=0.010** |
| T3 | **1.33 (1.10, 1.62), p=0.003** | 1.06 (0.89, 1.26), p=0.500 | 1.02 (0.86, 1.21), p=0.800 | **1.24 (1.02, 1.50), p=0.028** |
| Models adjusted for age (years) + sex (female, male) + ethnicity (“Non-Hispanic White”, “Hispanic”, “Black”) + educational level (years) + and cognitive status (“Normal cognition”, “Mild cognitive impairment”, “Dementia”) + cardiovascular diseases (binary) + hypertension (binary) + dyslipidemia-related classes (“No dyslipidemia”, “Dyslipidemia without medication”, “Dyslipidemia with medication”) + T2DM-related classes (“No diabetes”, “Diabetes without medication”, “Diabetes with medication”) + use of benzodiazepines (binary) + Alcohol consumption (binary) + current Tobacco smoking (binary). | | | | |
