# Supplementary material for: Networks and clusters of immunometabolic biomarkers and depression-associated features in middle-aged and older community-dwelling US adults with and without depression
Source: Brain Behav Immun Health. 2025 Sep 17;49:101103. doi: 10.1016/j.bbih.2025.101103 (PMC12523063; doi:10.1016/j.bbih.2025.101103)
Supplement: Multimedia component 8 [file mmc8.docx]

**Supplementary Table 8:** Regression Model 3 with independent variables classified in tertiles.

|  | Anhedonia and lack of motivation | Melancholia and negative emotions or cognitions | Worry and irritability | Cognitive complains |
| --- | --- | --- | --- | --- |
| IL- 6 | | | | |
| T1 | — | — | — | — |
| T2 | **1.40 (1.10, 1.79), p=0.007** | 1.10 (0.84, 1.43), p=0.500 | 0.91 (0.72, 1.16), p=0.400 | 1.02 (0.80, 1.30), p=0.900 |
| T3 | **1.45 (1.12, 1.89), p=0.005** | 1.24 (0.94, 1.62), p=0.120 | 0.99 (0.77, 1.28), p>0.900 | 1.19 (0.92, 1.56), p=0.200 |
| HbA1c | | | | |
| T1 | — | — | — | — |
| T2 | 0.87 (0.72, 1.06), p=0.200 | 0.93 (0.75, 1.14), p=0.500 | 0.92 (0.76, 1.11), p=0.400 | 1.09 (0.89, 1.33), p=0.400 |
| T3 | 1.14 (0.93, 1.40), p=0.200 | 1.15 (0.93, 1.41), p=0.200 | 0.89 (0.73, 1.08), p=0.200 | 1.06 (0.86, 1.30), p=0.600 |
| Abdominal circumference | | | | |
| T1 | — | — | — | — |
| T2 | 1.06 (0.88, 1.28), p=0.500 | 1.08 (0.89, 1.32), p=0.400 | 0.92 (0.76, 1.10), p=0.400 | 0.99 (0.82, 1.20), p>0.900 |
| T3 | **1.30 (1.06, 1.59), p=0.010** | 1.02 (0.84, 1.26), p=0.800 | 0.87 (0.72, 1.05), p=0.140 | 1.09 (0.89, 1.33), p=0.400 |
| BMI | | | | |
| T1 | — | — | — | — |
| T2 | 1.04 (0.86, 1.26), p=0.700 | 0.98 (0.80, 1.19), p=0.800 | 0.92 (0.77, 1.10), p=0.400 | **1.26 (1.04, 1.52), p=0.020** |
| T3 | **1.23 (1.01, 1.51), p=0.042** | 0.92 (0.75, 1.13), p=0.400 | 0.92 (0.76, 1.11), p=0.400 | 1.16 (0.95, 1.42), p=0.140 |
| Models adjusted for age (years) + sex (female, male) + ethnicity (“Non-Hispanic White”, “Hispanic”, “Black”) + educational level (years) + and cognitive status (“Normal cognition”, “Mild cognitive impairment”, “Dementia”) + cardiovascular diseases (binary) + hypertension (binary) + dyslipidemia-related classes (“No dyslipidemia”, “Dyslipidemia without medication”, “Dyslipidemia with medication”) + T2DM-related classes (“No diabetes”, “Diabetes without medication”, “Diabetes with medication”) + use of benzodiazepines (binary) + Alcohol consumption (binary) + current Tobacco smoking (binary) + depression-related classes (“No (current) depression”, “Current depression without medication”, “Current depression with medication”). | | | | |
